# Quantification of [^18^F]florbetapir: A test–retest tracer kinetic modelling study

**DOI:** 10.1177/0271678X18783628

**Published:** 2018-06-13

**Authors:** Sandeep SV Golla, Sander CJ Verfaillie, Ronald Boellaard, Sofie M Adriaanse, Marissa D Zwan, Robert C Schuit, Tessa Timmers, Colin Groot, Patrick Schober, Philip Scheltens, Wiesje M van der Flier, Albert D Windhorst, Bart NM van Berckel, Adriaan A Lammertsma

**Affiliations:** 1 Department of Radiology & Nuclear Medicine, Amsterdam Neuroscience, VU University Medical Center, Amsterdam, the Netherlands; 2 Department of Neurology & Alzheimer Center, Amsterdam Neuroscience, VU University Medical Center, Amsterdam, the Netherlands; 3 Department of Nuclear Medicine & Molecular Imaging, University of Groningen, University Medical Center Groningen, Groningen, the Netherlands; 4 Department of Anaesthesiology, Amsterdam Neuroscience, VU University Medical Center, Amsterdam, the Netherlands; 5 Department of Epidemiology & Biostatistics, Amsterdam Neuroscience, VU University Medical Center, Amsterdam, the Netherlands

**Keywords:** Amyloid positron emission tomography, Alzheimer’s disease, test–retest, [^18^F]florbetapir, tracer kinetic modelling

## Abstract

Accumulation of amyloid beta can be visualized using [^18^F]florbetapir positron emission tomography. The aim of this study was to identify the optimal model for quantifying [^18^F]florbetapir uptake and to assess test–retest reliability of corresponding outcome measures. Eight Alzheimer’s disease patients (age: 67 ± 6 years, Mini-Mental State Examination (MMSE): 23 ± 3) and eight controls (age: 63 ± 4 years, MMSE: 30 ± 0) were included. Ninety-minute dynamic positron emission tomography scans, together with arterial blood sampling, were acquired immediately following a bolus injection of 294 ± 32 MBq [^18^F]florbetapir. Several plasma input models and the simplified reference tissue model (SRTM) were evaluated. The Akaike information criterion was used to identify the preferred kinetic model. Compared to controls, Alzheimer’s disease patients had lower MMSE scores and evidence for cortical Aβ pathology. A reversible two-tissue compartment model with fitted blood volume fraction (2T4k_V_B_) was the preferred model for describing [^18^F]florbetapir kinetics. SRTM-derived non-displaceable binding potential (BP_ND_) correlated well (r^2 ^= 0.83, slope = 0.86) with plasma input-derived distribution volume ratio. Test–retest reliability for plasma input-derived distribution volume ratio, SRTM-derived BP_ND_ and SUVr_(50–70)_ were *r* = 0.88, *r = *0.91 and *r = *0.86, respectively. In vivo kinetics of [^18^F]florbetapir could best be described by a reversible two-tissue compartmental model and [^18^F]florbetapir BP_ND_ can be reliably estimated using an SRTM.

## Introduction

Accumulation of amyloid beta (Aβ) is one of the pathological hallmarks of Alzheimer’s disease (AD), which starts to accumulate years before clinical presentation of dementia.^1^ Aβ can be visualized using [^18^F]florbetapir positron emission tomography (PET). Accurate quantification of Aβ is important for patient management, monitoring progression of disease and response to disease-modifying therapies.^2–7^

Earlier studies with dynamic PET scans have shown increased [^18^F]florbetapir uptake in the cortex, especially precuneus and fronto-temporal regions, of patients with AD compared with healthy controls, presumably reflecting elevated accumulation of amyloid.^8,9^ To date, most studies have used the standardized uptake value ratio (SUVr) as semi-quantitative measure of [^18^F]florbetapir uptake. Test–retest studies, however, have generated inconsistent findings with regard to SUVr optimisation.^10–13^ SUVr may be too biased to identify near normal levels of amyloid deposition. More importantly, tracer kinetics and distribution are likely to be affected by underlying pathophysiological mechanisms, such as decreased perfusion known to occur in AD^16^. Effects of perfusion changes on bias in SUVr have already been documented for [^11^C]PiB.^14^

A validated tracer kinetic model is important, not only for identification of early (subtle) amyloid accumulation, but also for longitudinal assessment of changes in amyloid depositions.^14^ This is also the case for [^18^F]florbetapir, a widely used amyloid tracer, especially when it is used as a surrogate marker for assessing the efficacy of disease modifying drugs.^7^ Therefore, the main objective of this study was to identify the optimal tracer kinetic model for quantification of [^18^F]florbetapir binding, taking into account both test–retest reliability and optimisation of scan duration.

## Material and methods

### Participants

Eight patients with probable AD^3^ from the Amsterdam Dementia Cohort^17^ were included. Screening included physical and neurological examinations, medical history, extensive neuropsychological assessment, brain MRI, lumbar puncture and laboratory measurements (e.g. haemoglobin levels). AD patients were eligible when Mini-Mental State Examination (MMSE) scores were ≥19. Eight healthy control subjects were recruited through advertisements in newspapers. These controls were in good physical health, experienced no cognitive complaints and met Research Diagnostic Criteria for “never mentally ill.”^18^ Controls underwent a similar screening (except for lumbar puncture) as AD patients and were only eligible if results of all clinical assessments were within corresponding normal ranges. The study was approved by the local Medical Ethics Review Committee of the VU University Medical Center, and all subjects provided written informed consent, in line with the Helsinki Declaration of 1975 (and 1983 revised) guidelines.

### [^18^F]florbetapir synthesis

Individual doses of [^18^F]florbetapir were prepared on site in accordance with Avid Radiopharmaceuticals Investigational quality control release criteria.

### Data acquisition

Data were acquired using an Ingenuity TF PET/CT scanner (Philips Medical Systems, Best, the Netherlands). Prior to scanning, two cannulas were inserted, one for intravenous [^18^F]florbetapir administration and the other for arterial sampling. First, a low-dose computed tomography (CT) scan was performed for attenuation correction purposes. Each subject underwent two [^18^F]florbetapir PET scans (average interval: 4 ± 2 weeks). Following the low-dose CT, a 90-min PET emission scan was acquired after a bolus injection of 370 MBq (initial six scans) or 425 MBq (subsequent 26 scans) [^18^F]florbetapir. This increase in injected dose after six scans (three subjects) was introduced because of significant sticking of [^18^F]florbetapir to the wall of the injection catheter. Arterial blood was sampled continuously at a rate of 5 mL min^−1^ for the first 5 min and 2.5 mL min^−1^ thereafter, using an online detection system.^19^ At set times (5, 10, 20, 40, 60, 75 and 90 min), continuous withdrawal was interrupted briefly for the collection of manual blood samples (8 mL each), which were used to estimate plasma-to-whole-blood ratios and to measure plasma metabolite fractions.

For brain tissue segmentation and PET co-registration, structural MRI scans (3D T1-weighted using a magnetization-prepared rapid gradient-echo sequence) were acquired at 3.0 Tesla using either a Signa HDxt MRI (General Electric, Milwaukee, WI) or an Ingenuity TF PET/MR (Philips Medical Systems, Cleveland, OH) scanner.

### Radiometabolite analysis

Blood was collected in heparin tubes and centrifuged for 5 min at 5000 r/min. Plasma was separated from blood cells, and about 1 mL was diluted with 2 mL water and loaded onto a tC18 Sep-Pak cartridge (Waters, Milford, MA), which was pre-activated by elution with 6 mL of methanol and 12 mL of water, respectively. The cartridge was washed with 3 mL water to collect the polar radioactive fraction. Thereafter, the tC18 Sep-Pak cartridge was eluted with 2 mL of methanol and 2 mL of water to collect the mixture of non-polar metabolites. This fraction was further analysed by HPLC using an Ultimate 3000 system (Dionex, Sunnyvale, CA) equipped with a 1-mL loop. As a stationary phase, a Gemini C18, 250 × 10 mm, 5 µm (Phenomenex, Torrance, CA) was used. The mobile phase was a gradient of A = acetonitrile and B = 0.1% trifluoroacetic acid in water. The gradient ran for 15 min, decreasing the concentration of eluent B from 90% to 40% in 11 min, followed by 1 min of elution with 40% B at a flow rate of 4 mL min^−1^. The eluent was collected with a fraction collector (Teledyne ISCO Foxy Jr., Lincoln, NE), and the fractions were counted for radioactivity using a Wallac 2470 gamma counter (Perkin Elmer, Waltham, MA).

### Data analysis

PET images of 22 frames (1 × 15, 3 × 5, 3 × 10, 4 × 60, 2 × 150, 2 × 300 and 7 × 600 s) with a matrix size of 128 × 128 × 90 voxels and a final voxel size of 2 × 2 × 2 mm^3^ were reconstructed using 3D row action maximum likelihood algorithm. During reconstruction, all usual corrections for attenuation, scatter, randoms, decay and dead time were performed. Structural 3D T1-weighted MRI images were co-registered to the PET images. Using PVElab^20^ together with the Hammers template,^21^ regions of interest (ROIs) were delineated on the MRI scan and superimposed onto the dynamic PET scan to obtain regional time activity curves (TACs).

Using the information extracted from manual blood samples, online arterial blood TACs were calibrated and corrected for plasma to whole blood ratios, radiolabelled metabolites and delay, thereby generating individual metabolite corrected plasma input functions. Due to difficulties with blood data metabolite analysis (e.g. HPLC peak isolation, after four scan methods for peak separation were optimized, blood metabolite failed finally during n = 5 scans), missing values during online (continuous) detection of blood data within the first 5 min (n = 3 participants), and insufficient blood sample volumes (1 scan), plasma input-based pharmacokinetic test–retest analyses (using the original metabolite information) were performed for only five AD patients and four controls. There were six controls and eight AD patients from which we had blood data available from at least one satisfactory scan session and blood data. Because of the high intra-subject variability in parent fraction estimates, input functions were also derived using (1) a population average parent fraction (i.e. mean of all reliable parent fractions from all data sets) and (2) an intra-subject average parent fraction (i.e. mean parent fraction of test and retest scans). Parent fraction estimations are presented separately for controls and AD patients in Supplementary Figure 1, whilst Supplementary Figure 2 shows all three input functions for a representative control subject and an AD patient.

Several tracer kinetic models^22^ were used to fit the regional TACs, i.e. single tissue reversible (1T2k), and two tissue irreversible (2T3k) and reversible (2T4k) compartmental models, all with and without (V_B_) blood volume as additional fit parameter. The Akaike information criterion (AIC)^23^ was used to identify the optimal pharmacokinetic model for in vivo kinetics of [^18^F]florbetapir. In addition, the simplified reference tissue model (SRTM)^24^ and SUVr were assessed by comparing SRTM-derived non-displaceable binding potential (BP_ND_) and SUVr with distribution volume ratio (DVR). Cerebellar grey matter was used as reference region.

Test–retest (TRT) reliability of both microparameters (in particular the rate constant from blood to tissue K_1_) and macroparameters (distribution volume V_T_, DVR, BP_ND_ and SUVr_(50–70)_) was estimated for the preferred model and selected simplified methods. In addition, the impact of scan duration on model preferences, parameter values and TRT reliability was assessed. Finally, a separate comparison was made of parameter values for both controls and AD patients. This comparison was performed as an exploratory evaluation of the effects of the disease on tracer kinetics.

### Statistical analyses

Statistical analyses were performed using SPSS version 20.0.0 (IBM Corp., Armonk, NY). To investigate demographic, clinical and neuroimaging (i.e. SRTM-derived BP_ND_ group comparisons) data, χ^2^ tests for discrete variables and *t* tests for continuous data were used. Assumptions for normal distribution were checked using Kolmorogov–Smirnov tests. AIC was used to compare the model fits for regional TACs in order to identify the optimal tracer kinetic model. Standard deviations were used to evaluate the reliability of estimated parameters. TRT reliability was expressed as the Pearson correlation coefficient (r) of the parameter of interest between test and retest data, which was calculated for plasma input (K_1_, V_T_ and DVR) and SRTM (R_1_ and BP_ND_)-derived parameters.

## Results

Demographic and clinical data are presented in Table 1. Net injected dose and specific activity were comparable between groups, and between test and retest scans (all p values > 0.05). There were no differences in age, sex, body weight and length between patients with AD and controls (all p values > 0.05). MMSE scores were lower in AD patients than in controls (p < 0.001). Visual assessment of the [^18^F]florbetapir PET scans showed that all healthy controls showed no evidence of abnormal amyloid accumulation, whereas all patients with AD showed abnormal amyloid accumulation.

**Table 1.** Clinical and demographic data.

	Controls (n = 8)		AD patients (n = 8)		p value
Age	63.0 (4.4)		66.8 (5.9)		p = 0.17
Males/females (n)	3/5		3/5		NA
MMSE	29.8 (0.5)		22.6 (3.3)		p < 0.001
Body weight (in kg)	84.9 (14.6)		84.6 (12.3)		p = 0.97
Body length (in cm)	178.0 (12.9)		178.4 (8.7)		p = 0.95
	Test	Retest	Test	Retest	
Injected dose (MBq)	288 (42)	283 (36)	287 (39)	316 (10)	p^w ^= 0.24, p^b ^= 0.17
Specific activity (µg/mL)	3 (1)	4 (1)	3 (1)	4 (1)	p^w ^= 0.37, p^b ^= 0.87

Note: Data are presented as mean (SD).

BP_ND_: non-displaceable binding potential; p^w^: p value between test and retest measurements; p^b^: p value between AD and controls; SD: standard deviation; AD: Alzheimer’s disease; MMSE: Mini-Mental State Examination (range: 0–30); NA: not applicable.

**Figure 1.**
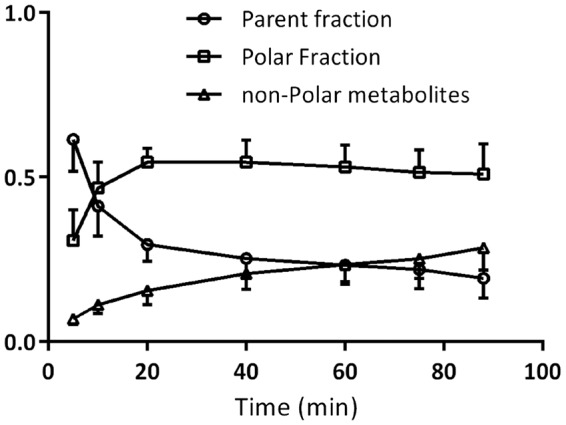
Parent [^18^F]florbetapir and polar metabolite fractions (mean ± SD) in arterial plasma at different time points.

**Figure 2.**
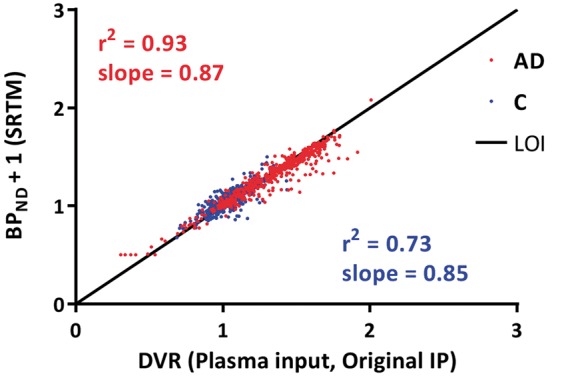
Comparison of SRTM-derived BP_ND_ against plasma input (Original IP)-derived DVR. Original IP is the input function obtained using original parent fractions. AD: Alzheimer’s disease; C: controls; DVR: distribution volume ratio; SRTM: simplified reference tissue model; BP_ND_: non-displaceable binding potential; IP: input; LOI: line of identity.

All plasma input-based kinetic analyses were performed on data of four controls and five AD patients. Tracer metabolism in plasma was relatively fast with parent fractions of about 60% and 20% after 5 - and 90-min post-injection (Figure 1), respectively. According to AIC, the 2T4k_V_B_ model described in vivo [^18^F]florbetapir kinetics best, irrespective of subject, ROI and type of input function. BP_ND_ (=k_3_/k_4_) values obtained using 2T4k_V_B_ did not correlate well with DVR-1, the indirect plasma input binding estimate (r^2 ^= 0.01, slope = 0.06). This most likely is due to poor precision (high standard deviations) of direct BP_ND_ estimates. For reference region-based analyses, data of eight controls and eight patients were used. There was a strong correlation between SRTM-derived BP_ND_ and plasma input DVR values (r^2 ^= 0.83, slope = 0.86 across all subjects, r^2 ^= 0.93 for AD, r^2 ^= 0.73 for controls; Figure 2).

**Figure 3.**
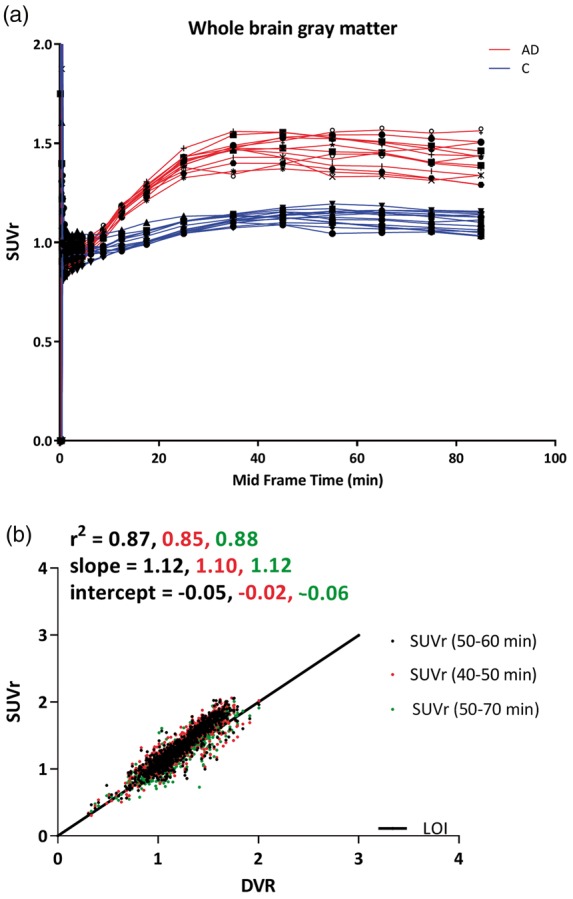
(a) Average whole brain grey matter SUVr values as function of time for each subject. Different symbols represent subjects (red lines are AD patients and blue lines are controls). (b) Comparison of SUVr obtained from data of different scan durations (40–50 min, 50–60 min and 50–70 min) against DVR, for all regions of interest included in the Hammers template. AD: Alzheimer’s disease; C: controls; DVR: distribution volume ratio; SUVr: standardized uptake value ratio.

Across subjects, different SUVr time intervals provided results that were comparable with DVR (original input function) for all ROIs. Figure 3 shows SUVr plots of whole brain grey matter together with a comparison of SUVr values obtained using different scan intervals with DVR (original input function) for all ROIs of all subjects. Irrespective of time intervals, SUVr overestimated (i.e. slope > 1) [^18^F]florbetapir binding when compared with DVR. Comparable correlation coefficients (∼r^2 ^= 0.86) and slopes (∼1.11) were observed for different SUVr time intervals (>40 min) for all subjects (Figure 3(b)), with substantially lower correlation coefficients for controls (e.g. SUVr_(50–70)_ r^2 ^= 0.61, slope = 0.88) compared to AD (e.g. SUVr_(50–70)_ r^2 ^= 0.88, slope = 1.11).

**Figure 4.**
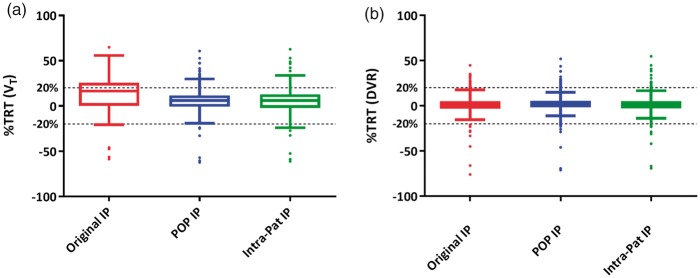
%TRT reliability of (a) V_T_ and (b) DVR observed in all Hammers template defined brain regions of both AD patients and controls, when using either of the three input functions. Original IP is the input function obtained using original parent fractions; POP IP is the input function obtained using population average parent fractions; Intra-pat IP is the input function obtained using intra subject average (test–retest) parent fractions. TRT: test–retest; DVR: distribution volume ratio.

A TRT reliability coefficient (r) of 0.75 was found for individual plasma input function-derived V_T_ values, with improved TRT reliability coefficients of 0.87 and 0.86 for V_T_ values obtained using population averaged and intra-subject averaged metabolite corrected plasma input functions, respectively. DVR, SRTM-derived BP_ND_ and SUVr_(50–70)_ showed TRT reliability coefficients of 0.88, 0.91 and 0.86, respectively. Figure 4 illustrates TRT reliability of V_T_ and DVR values across all volumes of interest for the three different types of input functions.

**Figure 5.**
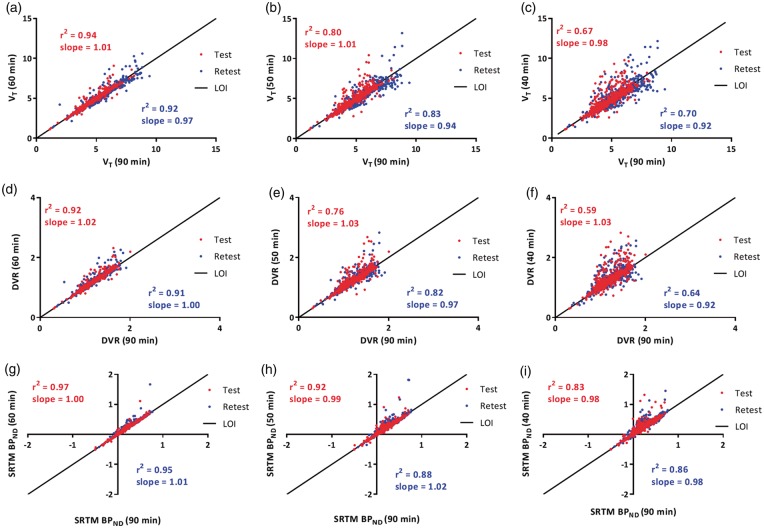
Impact of scan duration on kinetic parameter estimates: V_T_ (a, b and c), DVR (d, e and f), SRTM BP_ND_ (g, h and i). Reliability of estimated parameters decreased for shorter scan durations, similarly between test (red points) and retest (blue points) data. DVR: distribution volume ratio; LOI: line of identity.

Finally, Figure 5 shows the impact of overall scan duration on estimated values of V_T_, DVR and SRTM-derived BP_ND_. TRT reliability of the kinetic parameters obtained for different scan durations is provided in Table 2. When comparing AD patients with healthy controls, increased cortical SRTM BP_ND_ was found in temporal lobe (controls (mean ± SD) 0.02 ± 0.04, AD 0.31 ± 0.04, p < 0.001), frontal lobe (controls 0.08 ± 0.05, AD 0.43 ± 0.06, p < 0.001), occipital lobe (controls 0.19 ± 0.03, AD 0.34 ± 0.14, p = 0.07), parietal lobe (controls 0.02 ± 0.03, AD 0.23 ± 0.03, p < 0.001), posterior cingulate cortex (controls 0.23 ± 0.17, AD 0.55 ± 0.04, p = 0.004), but not in the hippocampus (controls 0.02 ± 0.05, AD 0.07 ± 0.05, p = 0.16).

**Table 2.** Kinetic parameters for different scan durations.

	90 min				60 min				50 min				40 min			
	AD		C		AD		C		AD		C		AD		C	
	TRT	Slope	TRT	Slope	TRT	Slope	TRT	Slope	TRT	Slope	TRT	Slope	TRT	Slope	TRT	Slope
DVR^a^	0.84	0.85	0.78	0.75	0.69	0.70	0.70	0.61	0.58	0.55	0.61	0.50	0.51	0.45	0.40	0.32
SRTM BP_ND_	0.87	0.90	0.91	0.87	0.83	0.91	0.90	0.89	0.74	0.87	0.82	0.76	0.76	0.80	0.79	0.71
SRTM R_1_	0.94	1.00	0.95	0.96	0.95	1.00	0.95	0.96	0.94	1.00	0.95	0.96	0.95	0.99	0.96	0.96
V_T_^a^	0.78	1.10	0.09	0.11	0.69	0.90	0.21	0.20	0.58	0.70	0.38	0.31	0.49	0.59	0.36	0.29
K_1_^a^	0.77	1.03	0.62	0.65	0.77	1.03	0.61	0.64	0.77	1.02	0.60	0.62	0.76	1.04	0.59	0.62

Note: TRT (r^2^) and slopes (60, 50 or 40 min) are based on comparisons with 90-min PET scans. Ninety-minute comparisons were done against 2T4k_V_B_ values.

TRT: test–retest; PET: positron emission tomography; AD: Alzheimer’s disease; C: controls; DVR: distribution volume ratio; SRTM: simplified reference tissue model; BP_ND_: non-displaceable binding potential.

^a^Obtained using 2T4k_V_B_ and input function processed using the individual measured parent fractions.

## Discussion

This study demonstrated that in vivo kinetics of [^18^F]florbetapir could best be described by a reversible two tissue compartmental model with blood volume parameter (i.e. 2T4k_V_B_). This model produced robust and consistent longitudinal results for all input functions, which did not vary between brain regions. Furthermore, of the reference tissue-based parameter estimates, SRTM-derived BP_ND_ showed least bias, whereas SUVr consistently overestimated [^18^F]florbetapir uptake. In addition, TRT reliability was poorer for SUVr than for BP_ND_.

The present findings with regard to the model preference are in line with another recent study,^25^ and additionally showed that model preference was independent of type of input function and scan duration. Kinetics of [^18^F]florbetapir appeared to be rather rapid in vivo, and model preference did not change when data were restricted to 40 min. Compared with 90-min scan duration, TRT reliability of kinetic parameters started to deviate for shorter scan durations. Interestingly, SRTM-derived BP_ND_, plasma input-derived DVR-1 and V_T_ correlated well with the corresponding 90-min parameter estimates, even for a scan duration of 50 min. Although, based on this comparison, scan duration could be shortened to 50 min, TRT values indicated that a scan duration of 60 min would be required to obtain reproducible SRTM-derived BP_ND_ and R_1_ values without substantial bias compared with corresponding 90-min estimates. Although plasma input-derived V_T_ and DVR for 50- and 60-min data were comparable with corresponding 90-min estimates, the overall reproducibility was poorer than for reference tissue-based parameter estimates.

Unfortunately, isolation of HPLC peaks was unsatisfactory during the first four studies, and V_T_ obtained from the 2T4k_V_B_ model was affected by inaccuracies in parent fraction estimations. Initially, the temporal resolution of offline radioactivity detection (30-s fractions) was not sufficient to separate metabolite peaks on HPLC. To circumvent this problem, more fractions were collected around the two peaks that were present. Although this improved accuracy, at the same time count rates for each fraction were lower, reducing precision and, consequently, less reproducible parent fraction estimates. Unreliable parent fractions will, in turn, result in unreliable kinetic parameter estimates. For this reason, intra-subject averaged and population averaged parent fractions were investigated, and they resulted in better TRT of V_T_ values compared with those obtained with individually measured parent fractions. A TRT of less than 5% was observed for DVR and SRTM-derived BP_ND_, indicating that the probable reason for the relatively high TRT of V_T_ was indeed due to the error in parent fraction estimates. Interestingly, even when using population/intra-subject averaged parent fractions, the TRT of V_T_ was as high as 20%. One of the reasons for this might be that the use of a population average does not account for genuine inter-subject differences in tracer metabolism.

A point of concern is whether non-polar metabolites enter the brain and affect tissue kinetics. Non-polar metabolites were not characterized in this study and hence their nature is not known. However, in case of influx of non-polar metabolites in the brain, a slow and gradual increase in the uptake would be anticipated. This should lead to an irreversible nature in the regional TACs particularly for regions devoid of amyloid load, which was not observed for any ROI in this subject group. On the other hand, tissue TACs were well described by the 2T4k_V_B_ model, irrespective of the subject status, region size or the underlying amyloid load. Although, this is not absolute proof that non-polar metabolites do not enter the brain, it suggests that substantial influx of non-polar metabolites does not occur, at least not within the time frame of the scan.

SRTM BP_ND_ values corresponded well with plasma input DVR values, with the strongest correspondence found for AD patients. This could be explained by the observation that all AD patients in this study showed evidence of abnormal amyloid accumulation. Notwithstanding, for controls, there was also a good correspondence between plasma input DVR and SRTM BP_ND_, which suggests that SRTM can provide reproducible findings even in cases with lower or limited amounts of cortical Aβ. As expected, increased SRTM BP_ND_ values were found in AD patients compared with controls in all cortical lobar regions except the occipital lobe.^9^ Reproducible TRT values were found for SRTM with a minimum of 60-min scan duration. Therefore, SRTM should be the method of choice provided the reference tissue is not affected by amyloid disposition. Future studies should investigate which parametric method can be used for visual interpretation of [^18^F]florbetapir.

Despite a consistent overestimation, SUVr correlated well with plasma input-derived DVR. In a recent study,^25^ SUVr was validated against V_T_. This comparison, however, suffered from the fact that both V_T_ and SUVr depend on signals from free, specifically bound and non-specifically bound tracer. To assess whether SUVr is a good measure of amyloid load, it is necessary to validate it against BP_ND_, which only represents specific binding. The most commonly used SUVr time interval for [^18^F]florbetapir is 50- to 70-min post-injection.^8,9^ In this study, SUVr became constant from about 40 min onwards and, accordingly, a constant overestimation of approximately 10% compared with DVR was observed, independent of actual scan duration (>40 min), with substantially poorer performance in controls compared to AD. This suggests that, at least for the subjects included in this study, SUVr using a 40 - to 50-min scan interval could be sufficient as a semi-quantitative measure of amyloid load. Nevertheless, further studies in larger patient cohorts are needed to assess whether the bias in SUVr is indeed constant. In addition, simulation studies are required to assess whether the bias in SUVr varies with physiologically relevant changes in perfusion.

One limitation of a reference region approach is the potential presence of amyloid in the reference region, in this case grey matter cerebellum, as this would result in underestimation of the specific signal from other regions. The only way to confirm that a reference region is completely devoid of specific binding is by performing a pharmacological blocking study, but for amyloid tracers, this is not possible in humans. Considering that essentially no specific binding is present in grey matter cerebellum of controls, an alternative is to evaluate whether there is any difference in grey matter cerebellum V_T_ between AD patients and controls. Such a difference was not seen, indicating that grey matter cerebellum can be used as a reference region. Nevertheless, further studies are needed to validate the use of grey matter cerebellum as reference region.

## Conclusions

The 2T4k_V_B_ model is the preferred plasma input model for describing in vivo kinetics of [^18^F]florbetapir in both healthy controls and AD patients. Unfortunately, V_T_ seems to be affected by uncertainties in parent fraction estimates. Therefore, when a reliable reference region exists, SRTM is the preferred method of analysis, as it is both the most reliable and the most reproducible method, and scan duration can be reduced to 60 min.

## Supplemental Material

Supplemental material for Quantification of [^18^F]florbetapir: A test–retest tracer kinetic modelling study Click here for additional data file. Supplemental material for Quantification of [^18^F]florbetapir: A test–retest tracer kinetic modelling study by Sandeep SV Golla, Sander CJ Verfaillie, Ronald Boellaard, Sofie M Adriaanse, Marissa D Zwan, Robert C Schuit, Tessa Timmers, Colin Groot, Patrick Schober, Philip Scheltens, Wiesje M van der Flier, Albert D Windhorst, Bart NM van Berckel and Adriaan A Lammertsma in Journal of Cerebral Blood Flow & Metabolism
