# Supplementary material for: Quantification of [18F]florbetapir: A test–retest tracer kinetic modelling study
Source: J Cereb Blood Flow Metab. 2018 Jun 13;39(11):2172–80. doi: 10.1177/0271678X18783628 (PMC6826855; doi:10.1177/0271678X18783628)
Supplement: Supplemental material for Quantification of [18F]florbetapir: A test–retest tracer kinetic modelling study [file Supplemental1_material.pdf]

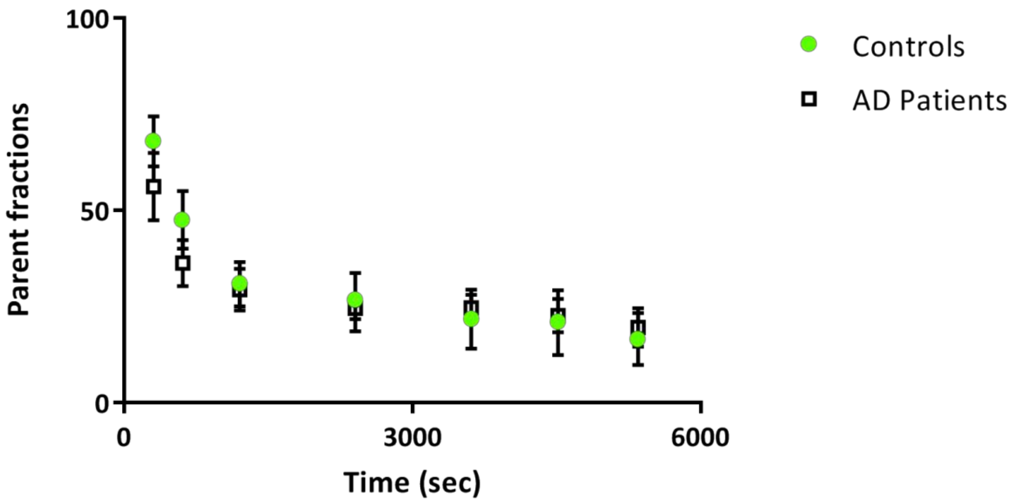

Supplementary figure 1. Parent fractions (mean ± SD) for AD patients and controls separately.

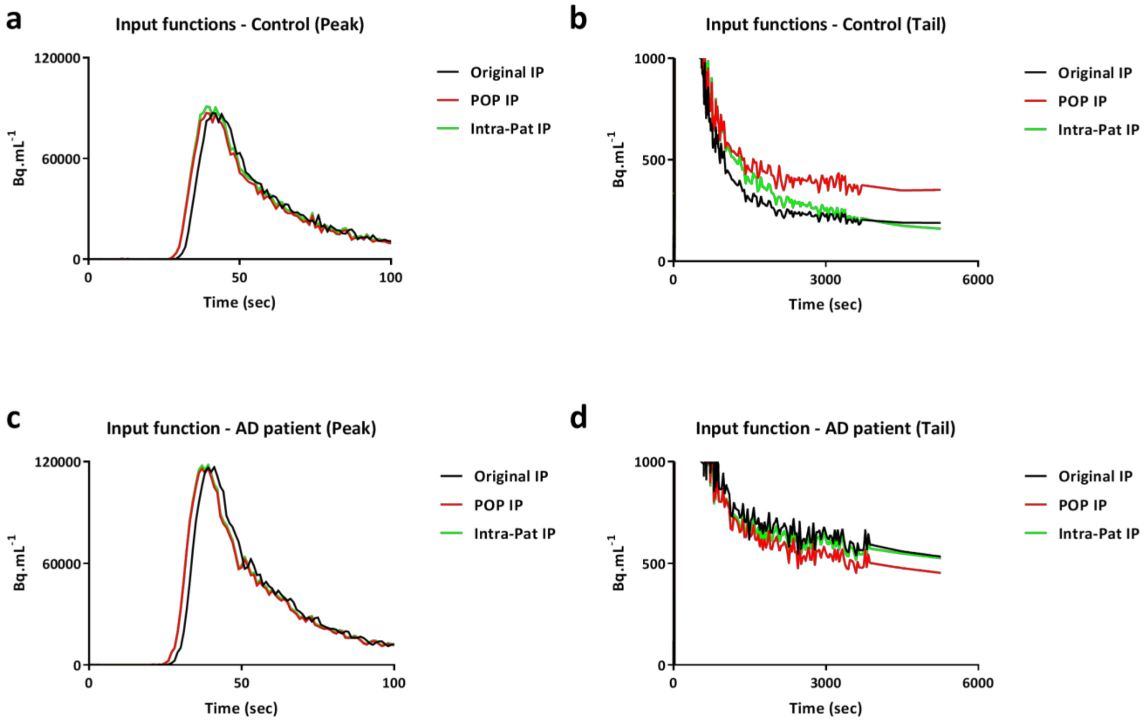

Supplementary figure 2. Three metabolite corrected plasma input functions (Original IP, POP IP, and Intra-Pat IP) for a representative control (**a**: peak, **b**: tail) and an AD patient (**c**: peak, **d**: tail). Original IP is the input function obtained using original parent fractions; POP IP is the input function obtained using population average parent fractions; Intra-pat IP is the input function obtained using intra subject average (test-retest) parent fractions.
